# Frequency of Celiac Disease in Patients With Chronic Diarrhea

**DOI:** 10.7759/cureus.20495

**Published:** 2021-12-17

**Authors:** Muhammad S Panezai, Asad Ullah, Kalyani Ballur, Lauren Gilstrap, Jaffar Khan, Bisma Tareen, Mirwais Kakar, Javeria Khan, Amna Rasheed, Abdul Waheed, Intisar Ghleilib, Joseph White, Frederick D Cason

**Affiliations:** 1 Gastroenterology, Bolan Medical College, Quetta, PAK; 2 Pathology, Medical College of Georgia, Augusta University, Augusta, USA; 3 Clinical Oncology, Medical College of Georgia, Augusta University, Augusta, USA; 4 Pathology and Laboratory Medicine, Indiana University School of Medicine, Indianapolis, USA; 5 Internal Medicine, Bolan Medical College, Quetta, PAK; 6 Gastroenterology, Holy Family Hospital, Rawalpindi, PAK; 7 Internal Medicine, Touro University California, Vallejo, USA; 8 Surgery, San Joaquin General Hospital, French Camp, USA; 9 Pathology and Laboratory Medicine, Medical College of Georgia, Augusta University, Augusta, USA

**Keywords:** prevalence, diagnosis, gluten intolerance, celiac disease, chronic diarrhea

## Abstract

Introduction: Celiac disease (CD) is an immune-mediated disease caused by ingesting gluten-containing foods and is characterized mainly by malabsorptive diarrhea. Furthermore, distinguishing between mild disease and asymptomatic individuals is critical and necessitates a high level of clinical suspicion. Short stature, delayed puberty, bone abnormalities, neurological problems, and intestinal cancer can all be consequences of a delayed diagnosis. This study aimed to determine the prevalence of celiac disease among our community's recurrent diarrhea patients.

Methods: This was a cross-sectional study aimed at determining the frequency of celiac disease in patients with chronic diarrhea. One hundred eighty-eight patients between the ages of 18 and 60 years who had chronic diarrhea lasting greater than three months were enrolled in this study. Stratification was utilized to control for modifiers. A p-value of ≤ 0.05 was considered significant.

Results: A total of 74.5% of patients (n=140) were male, while 25.5% (n=48) were female with a mean age of 38.48±10.85 years. The average duration of celiac disease symptoms was 8.17± 3.75 months. Celiac disease was found in 12.2% (n=23) of the individuals. Also, 21% of individuals with a positive family history of CD devolved CD, compared to those without prior CD family history (p=0.01).

Conclusions: In individuals with chronic diarrhea for more than three months, the prevalence of celiac disease was determined to be 12.2% (n=23). There was a statistically significant difference between those with a positive family history of CD and those who did not have the condition.

## Introduction

Celiac disease (CD) is an autoimmune disease characterized by gluten intolerance in patients with a genetic predisposition [1]. Gluten is found in wheat, rye, barley, spelt, and kamut [1]. Gluten can cause diarrhea, bloating, stomach pain, and weight loss in those with CD [1]. CD causes inflammatory damage to the small intestinal mucosa, resulting in villous atrophy and crypt hyperplasia, promoting malabsorption [2]. Micronutrient deficits such as vitamin B12, fat-soluble vitamins, folic acid, and iron can be caused by malabsorption [2]. Inflammatory damage, which increases water and solute outflow, can cause diarrhea [3].

CD can manifest in various clinical features, making it challenging to identify solely on symptoms alone. Iron deficiency anemia and irritable bowel syndrome are two of the most prevalent clinical symptoms, even though they are non-specific [4,5]. The frequency of CD was 6.5-21% among patients with chronic diarrhea in the Middle East and North Africa, according to Barada et al., with gastrointestinal symptoms being the most common symptom [6]. Rampertab et al. looked at CD presentation trends in the United States (US) from 1952 to 2004 and found that diarrhea was present in 46.7% of their sample, but the trend for diarrhea as a presenting symptom was shifting downward [7].

This study aimed to determine the frequency of CD in chronic diarrhea patients at a tertiary care hospital. Since chronic diarrhea is a common complaint with many etiologies, understanding how frequently this is a manifestation of CD will aid physicians in making quicker diagnoses and initiating treatment. Through this study, we will expand on literature outlining the many presentations of celiac disease by focusing on chronic diarrhea and its prevalence.

## Materials and methods

A strategy for evaluating the incidence of celiac disease in individuals with persistent diarrhea was developed to be evaluated by institutional review boards (IRBs). Following a thorough review of the protocol, the IRB team gave its official approval for the study to proceed. There were 188 patients who participated in a cross-sectional single-center study that was carried out in an academically tertiary care hospital. Males and females between the ages of 18 and 60 years who had chronic diarrhea that had lasted more than three months were eligible to participate in the study. Exclusion criteria included patients having blood in their stool as well as those suffering from other autoimmune disorders, such as autoimmune hepatitis, multiple sclerosis, and autoimmune thyroid problems.

There were a total of 188 participants in this study who met the inclusion criteria and provided their consent after being informed. It was necessary to collect 10 mL blood samples for an IgA anti-tissue transglutaminase antibody test or for endoscopic duodenal biopsies. The lab reports were evaluated by a pathologist consultant. SPSS software version 21 (Armonk, NY: IBM Corp.) was used to examine the data. The mean and standard deviation of quantitative data were calculated, while the frequencies and percentages of qualitative variables were calculated.

Following the stratification of the data, the chi-square test was utilized to conduct the analysis. A p-value of 0.05 or lower was considered statistically significant. During our research, data were stored on secure network encrypted drives in a password-protected office at our institution. Only approved individuals were granted access to the data in this study, and each of these users was assigned a unique password to protect their information. Once the project was completed, the data were deleted from the system.

## Results

Demographical and underlying comorbid conditions

The participants' average ages were 38.48 years and 10.85 years, respectively, with 74.5% (n=140) male and 25.5% (n=48) female. Diabetes mellitus was determined to be the underlying comorbid condition in 2.7% (n= 5) of the patients, while 33% (n=62) of the patients had a positive family history of celiac disease, indicating that the condition may have a hereditary influence (Table 1).

**Table 1:** Demographical distribution of patients in the study DM: diabetes mellitus; CD: celiac disease; N: numbers

Demographics		Frequency (N)	Percentage (%)	Mean age (Y ±SD)
Gender	Male	140	74.5	38.57 ± 10.96
	Female	48	25.5	38.21 ± 10.63
	Total	188	100.0	38.48 ± 10.85
History of DM	Positive	05	2.7	2.7
	Negative	183	97.3	100.0
	Total	188	100.0	
Family history of CD	Positive	62	33.0	33.0
	Negative	126	67.0	100.0
	Total	188	100.0	

Frequency of CD and age groups

A total of 23 individuals in the overall cohort tested positive for CD, accounting for 12.2% of the total (Table 2). CD was found to be more prevalent in patients in the age groups of 36-45 years, followed by the age groups of 18-35 years, and 46-60 years, even though the difference was statistically insignificant (p=0.533) (Table 3).

**Table 2:** Frequency of celiac disease in the selected population CD: celiac disease; N: number

CD	Frequency (N, %)	Cumulative percentage (%)
Positive	23 (12.2)	12.2
Negative	165 (87.8)	100
Total	188 (100)	100

**Table 3:** Comorbidities, family history, and duration of symptoms of CD CD: celiac disease; N: numbers

Celiac disease		Positive (N, %)	Negative (N, %)	Total (N, %)	p-Value
Gender	Male	19 (13.6)	121 (86.4%)	140 (100)	0.339
	Female	4 (8.3)	44 (91.7)	48 (100)	
Age group (year)	18-35	9 (11.4)	70 (88.6)	79 (100)	0.533
	36-45	8 (16.7)	40 (83.3)	48 (100)	
	46-60	6 (9.8)	55 (90.2)	61 (100)	
History of diabetes mellitus	Present	1 (20)	4 (80)	5 (100)	0.591
	Absent	22 (12)	161 (88)	183 (100)	
Family history of CD	Present	13 (21)	49 (79)	62 (100)	0.010
	Absent	10 (7.9)	116 (92.1)	126 (100)	
Duration of symptoms of CD	<6 months	7 (8.5)	75 (91.5)	82 (100)	0.170
	6-12 months	8 (11.9)	59 (88.1)	67 (100)	
	>12 months	8 (20.5)	31 (79.5)	39 (100)	

Frequency of diarrhea data

Patients began to exhibit CD symptoms on average after 8.17 months, with a mean standard deviation of 3.75 months. The age distribution of the population as a whole, broken down by gender, is shown in Table 1. Moreover, the data related to the frequency of the CD in relation to various variables including gender, age, comorbidities are summarized in Table 2 and Table 3. More detailed breakdowns of the key components, as well as the duration of symptoms, are provided in Table 3. There was a statistically significant difference between patients with CD and those who had a family history of CD. There appears to be no correlation between CD and any other characteristic.

## Discussion

CD was once assumed to be a disease that only affected Caucasian Europeans, but it is now found in people worldwide [8]. CD is found in 0.5-1% of the population worldwide [8]. Africa, the Middle East, Asia, and South America all have reported underdiagnosis of CD [8]. The influence of a Western diet has led to an increase in the intake of gluten-containing foods in Asia, such as bread and noodles, which could increase the prevalence of CD in the future [9]. The HLA-DQ2 and HLA-DQ8 genotypes are found in most CD patients [8]. Immune cells are exposed to these chemicals, which results in immune-mediated small intestine enteropathy and malabsorption [9].

CD can cause gastrointestinal symptoms such as diarrhea, steatorrhea, and weight loss, as well as extraintestinal symptoms such as anemia, osteoporosis, and dermatitis herpetiformis, or it can go unnoticed [8]. CD presentation is diverse because of the interaction of genetics, immunological factors, and the environment [8]. CD is more common in type 1 diabetes, autoimmune thyroiditis, autoimmune liver disease, Down syndrome, Turner syndrome, and Williams syndrome [10]. Microalbuminuria is associated with type 2 diabetes and can lead to kidney failure [11]. Patients with CD who have a first- or second-degree relative with the disease are more likely to get it themselves [10]. CD prevalence in children was 0.32-1.41% and in adults was 0.05-1.22% according to the Ashtari et al.'s thorough analysis of CD prevalence throughout the Asia-Pacific region [12].

Due to a lack of knowledge of the disease and consideration of serologic tests, a CD diagnosis can be missed [1]. CD can have a variety of clinical manifestations that vary from patient to patient [13]. As a result, a lack of understanding and awareness of these symptoms can delay diagnosis and testing [13]. Although the frequency of CD diagnoses has increased in the previous 30 years, many people remain undiagnosed [1]. There are a variety of recommendations for diagnosing CD. In patients above the age of two years, the American College of Gastroenterology recommends IgA anti-tissue transglutaminase antibody testing and intestinal biopsy in patients with a strong suspicion of CD [14]. According to the British Society of Gastroenterology, serological tests and duodenal biopsy are required for the diagnosis of CD [15]. According to the World Gastroenterological Association, anti-tissue transglutaminase (anti-tTg) IgA test and endoscopy are sufficient for diagnosis in low-resource countries, but intestinal biopsy is indicated in places with qualified pathologists [16]. Although these guidelines differ significantly, they agree on serological testing and biopsy when it is possible.

Furthermore, from 1952 to 2004, Rampertab et al. investigated the trends in the clinical presentation of CD patients detected by biopsy at one center [7]. The participants were divided into five groups based on their diagnosis, childhood diagnosis, disease duration, clinical presentation, and tumor presence [7]. At the time of diagnosis, the average age was 43.3 years and 17.4 years. CD ran in the family for 25.1% of the patients [7]. From the onset of symptoms to diagnosis, the average time was 4.67.5 years [7]. In 46.7% of patients, diarrhea was the predominant symptom [7]. Over time, there was a negative linear trend in the fraction of those who presented with diarrhea [7]. Also, diarrhea was detected in 91.3% of patients before 1981, while 37.2% were diagnosed after 2000 [7]. There was no statistically significant difference in age at presentation [7]. Before diagnosis, there was no statistically significant reduction in the length of diarrheal symptoms, although there was a statistically significant reduction in the duration of symptoms in patients who did not have diarrhea [7]. This could indicate that doctors are more aware of other CD symptoms but do not consider CD in patients who report diarrhea [7]. In our study, 12.2% of the patients with chronic diarrhea were tested positive for CD.

Furthermore, Green, in a study to determine the wide range of clinical symptoms that CD patients experience, conducted a large-scale study [17]. Two hundred twenty-seven patients with biopsy-proven CD were seen at Columbia University's Celiac Center in New York for the study [20]. The disease was found to be more prevalent in the female gender with an average age at diagnosis was 46.4 years ±1 year [17]. The duration of symptoms decreased from 9±1 years to 4.4±6 years before and after 1993 [17]. In approximately 62% of people, diarrhea was the most prevalent symptom [17]. Before 1993, when serologic testing was first performed, 73% of diarrhea cases were diagnosed, while only 43% were diagnosed after [17].

Previous research and this one have shown that diarrhea is a common symptom of CD. This study is noteworthy because it reports the prevalence of CD in rural areas with limited access to healthcare facilities, which is underrepresented in the literature. The fact that a small group was evaluated quickly is a limitation of this study. More studies should be done with a larger sample size.

## Conclusions

Celiac disease was identified in 12.2% of individuals who had chronic diarrhea for more than three months. The frequency of this condition is investigated in this study in relation to underserved ethnic and regional characteristics. Diarrhea is a symptom of CD, so physicians should be aware of it and not rule it out entirely. It will be easier to detect and treat CD in its early stages and prevent long-term complications.
